# “My Voice Sounds Different”: A Case of Acute Idiopathic Velopharyngeal Insufficiency

**DOI:** 10.7759/cureus.74010

**Published:** 2024-11-19

**Authors:** Rita Aldeia da Silva, Sandra Catarina Ferraz, Inês Barros Rua, Sandrina Martins, Helena Ramalho

**Affiliations:** 1 Pediatrics, Hospital de Braga, Braga, PRT; 2 Pediatrics, Unidade Local de Saúde do Alto Minho, Viana do Castelo, PRT; 3 Pediatrics, Hospital Pediátrico de Coimbra, Coimbra, PRT

**Keywords:** acute, children, idiopathic, nasal regurgitation, rhinolalia, velopharyngeal insufficiency

## Abstract

This report details a case of acute idiopathic velopharyngeal insufficiency in a previously healthy eight-year-old girl, presenting with sudden voice alteration and nasal regurgitation following mild respiratory symptoms. Physical examination identified unilateral velar paralysis with open rhinolalia, without additional neurological deficits. Extensive diagnostic evaluation, including nasopharyngoscopy, cerebral and cervical imaging, and infectious serologies, yielded unremarkable findings. In the absence of spontaneous improvement after one week, the patient received a brief course of prednisolone and was referred for speech therapy, leading to full resolution of symptoms within one month. Acute idiopathic velopharyngeal insufficiency is an infrequent condition in pediatric populations, commonly associated with viral infections and typically self-limiting. This case underscores the necessity of ruling out alternative etiologies and maintaining follow-up to confirm the benign trajectory of the disorder.

## Introduction

Normal speech depends on proper velopharyngeal closure; any disruption can lead to hypernasal resonance and altered sound production [1].

The velopharyngeal complex is anatomically defined by the soft palate and oropharyngeal walls, forming a functional port between the oropharynx and the nasopharynx [2]. This complex plays a crucial role in nasal breathing, swallowing, and especially in speech [3]. This is facilitated by the coordinated action of multiple muscle groups, innervated by three different cranial nerves: the trigeminal, glossopharyngeal, and vagus nerves [4]. The vagus nerve, particularly its motor branches from the pharyngeal plexus, plays a significant role in the motor control of the soft palate, underscoring its importance in these vital functions [5].

Different terms have been used to describe the causes of dysfunction of this complex, leading to some confusion in the literature [1]. Velopharyngeal dysfunction is defined as any condition where the velopharyngeal valve fails to close adequately during oral sound production, and can be divided into three main categories: velopharyngeal mislearning, velopharyngeal incompetence, and velopharyngeal insufficiency (VPI), each requiring specific treatment approaches [1,6].

Velopharyngeal mislearning occurs when a child produces sounds in the pharynx instead of the mouth, causing airflow through the nose and mimicking VPI. Velopharyngeal incompetence, in contrast, arises from poor movement of the velopharyngeal structures, often linked to brain or cranial nerve disorders like cerebral palsy or traumatic brain injury [1]. Finally, VPI is a condition that impairs the proper closure of the velopharyngeal port during the speech, leading to nasal regurgitation and characteristic speech deficits such as hypernasal speech (rhinolalia), nasal emissions, and reduced speech intelligibility [2,7,8].

VPI is primarily a pediatric condition, most commonly congenital and often associated with craniofacial malformations [9]. Cleft palate is the leading cause of VPI in children, and even after early and successful palatoplasty, VPI persists in 30% of cases [10]. Other common congenital causes include hereditary myopathies, trisomy 21, 22q11 deletion syndrome, and the Pierre Robin sequence [1-3,9]. Acquired VPI can occur following procedures like adenoidectomy or tonsillectomy, or as a consequence of neurological conditions like ischemia, demyelinating diseases, or brain lesions [2,3,9]. In contrast to these forms, sudden-onset acute idiopathic VPI is a rare and less frequently reported condition, predominantly affecting children [4]. This condition often presents abruptly and is occasionally linked to recent viral infections, similar to other transient cranial mononeuropathies such as Bell's palsy, although its exact etiology remains unclear [2-4].

Approximately, only 40 cases of acute idiopathic VPI have been reported, with a male predominance. The left side is most frequently affected, while bilateral cases are extremely rare [2,4].

The aim of this report is to present a case of acute idiopathic VPI in a previously healthy child. It details the clinical presentation, diagnostic process, and management, and compares this case with similar cases reported in the literature to better understand this unusual disorder.

## Case presentation

A previously healthy eight-year-old girl presented to the emergency department with a sudden onset of voice changes, first noticed by her and her parents two days earlier. The parents described her voice as resembling a 'cartoon voice.' The patient also reported a fear of ingesting liquids following an episode of choking on water that led to nasal regurgitation. She had mild respiratory symptoms, including cough and rhinorrhea, but no fever. Additionally, she mentioned a mild headache without any alarming signs. Trauma or ingestion of a foreign body was ruled out.

**Figure 1:**
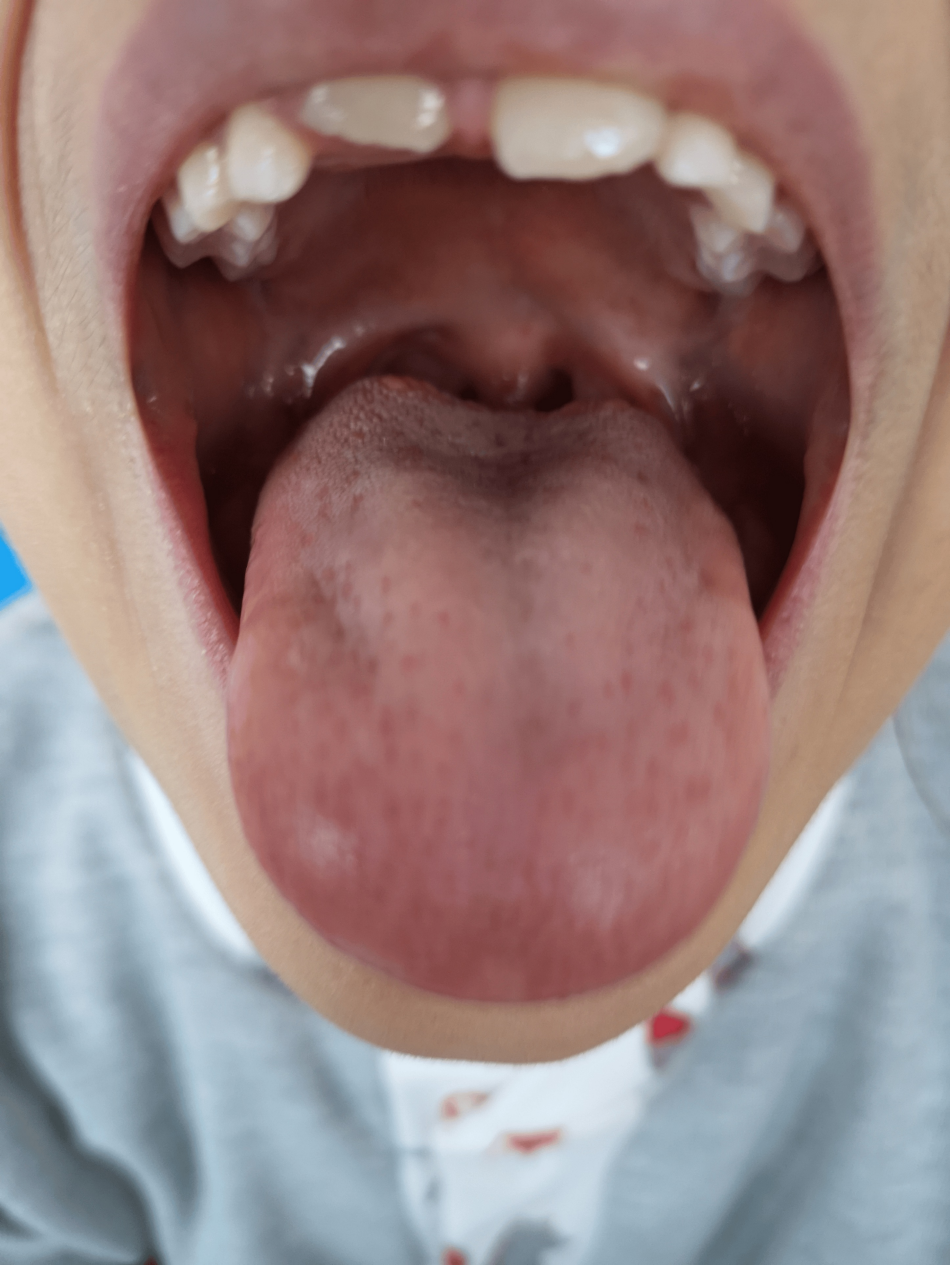
Oral examination Leftward uvular shift during contraction (phonation) caused by right unilateral velar paralysis.

On physical examination, she exhibited right-sided unilateral velar paralysis, resulting in hypernasal speech (open rhinolalia) during phonation, and the remaining neurological exam was unremarkable. There were no signs of skin rash or other neurological deficits. The gag reflex was intact, and pharyngo-laryngeal and lingual mobility were within normal limits (Figure 1). The remaining examination findings were normal. 

The complete blood count, biochemical profile, and muscle enzymes were all within normal limits. The erythrocyte sedimentation rate was 9 mm and C-reactive protein was negative. Infectious serologies for hepatitis A, cytomegalovirus, herpes simplex virus, varicella-zoster virus, parvovirus B19, and coxsackie virus were negative. Additionally, the respiratory panel, including tests for *Mycoplasma pneumoniae*, *Chlamydia pneumoniae*, and *Bordetella pertussis*, yielded negative results. *Borrelia burgdorferi* testing was also performed and returned negative.

A fiberoptic nasopharyngoscopy was performed to assess the involved muscles, which confirmed the diagnosis of VPI. To further investigate the cause, cervical and brain computed tomography (CT) scans, as well as magnetic resonance imaging (MRI) of the brain, were conducted. Both imaging studies were unremarkable, with no abnormalities detected in the intracranial portion of the glossopharyngeal nerve (cranial nerve IX) and the vagus nerve (cranial nerve X).

Despite the absence of spontaneous recovery after one week, the patient was treated with a five-day course of prednisolone and was referred for speech therapy. Over the next two weeks, her symptoms gradually improved, and she achieved complete resolution of the VPI within a month. At a follow-up visit three weeks later, the patient had fully recovered, with no recurrence of symptoms.

## Discussion

Acute idiopathic VPI is a rare and typically benign condition that predominantly affects children, characterized by a sudden onset and a generally favorable prognosis [11]. Since its initial description by Edin et al. in 1976 [12], approximately 40 cases have been reported in the literature [2,13].

Most cases occur in children between the ages of five and 15 years, typically around eight to nine years old, which is consistent with the age of the patient in this case. According to Walter et al., 73% of the affected children were male, while 27% were female [2]. In pediatric cases, VPI was consistently unilateral, although a few cases of bilateral VPI have been reported in adult women [3,4]. In contrast to the case described, the left side is more commonly affected in unilateral cases [4].

The clinical presentation typically included acute-onset hypernasal speech (rhinolalia) and nasal reflux in the majority of cases, as we see in this case [2,14]. Dysphagia was also commonly reported, with symptoms such as odynophagia, headache, and dyspnea occasionally observed. Additionally, a recent history of febrile illness, such as respiratory or gastrointestinal infections, was noted in about 25% of cases [4,11]. On physical examination, unilateral paralysis of the soft palate and a reduced gag reflex are commonly detected, though the patient’s overall condition typically remains stable [4,14].

The etiology of this disorder remains uncertain, though a viral origin has been postulated in some cases. The literature suggests that viral infections, particularly neurotropic viruses like coxsackie, parvovirus B19, and hepatitis A, might play a role, although direct evidence linking these infections to VPI is limited [3,14]. An immunologically mediated response, especially in cases involving non-neurotropic viruses, or a delayed inflammatory process, is also considered as a potential mechanism [4,11]. In the case presented, none of the agents commonly linked to acute idiopathic VPI were identified.

The diagnosis of idiopathic velopharyngeal paralysis is one of exclusion. While direct visual examination and fiberoptic nasopharyngoscopy can reveal muscle involvement, they do not identify the specific cranial nerves affected - glossopharyngeal (IX) or vagus (X) nerve [2-4]. MRI plays a crucial role in ruling out lesions in the posterior fossa and brainstem, as well as demyelinating or vascular pathologies, thereby helping to exclude more serious conditions. Although imaging studies, including MRI and CT scans, typically reveal no abnormalities, this lack of findings reinforces the idiopathic nature of the condition [2,14]. This supports the view that acute VPI is often a self-limiting condition with a favorable outcome, especially when no underlying neurological disorder is detected.

Regarding treatment, 89% of patients received no intervention, while 11% were treated with prednisolone (oral or intravenous) [2]. The prognosis is excellent, with most cases following a self-limiting course and achieving complete recovery in over 85% of cases within 2-3 weeks [14]. These findings suggest a likely viral origin, akin to Bell’s palsy, though the exact cause remains uncertain [11]. Treatment is mostly symptomatic, as the condition often resolves spontaneously over weeks or months. The favorable response to corticosteroids and speech therapy in this case supports early intervention, though further research is needed to clarify the pathophysiology and refine treatment approaches. Follow-up is essential to rule out progressive conditions like brainstem neoplasms or inflammatory diseases [2-4].

The case presented here contributes to the understanding of acute idiopathic VPI by reinforcing the importance of comprehensive diagnostic evaluation, including imaging studies and nasopharyngoscopy, to rule out structural abnormalities and progressive neurological conditions. The patient’s favorable response to corticosteroids and speech therapy also suggests that early intervention can be beneficial, even in idiopathic cases. However, the exact pathophysiology remains elusive, and further studies are necessary to establish more definitive management protocols.

## Conclusions

In conclusion, acute idiopathic VPI, although rare, is an important differential diagnosis in children presenting with sudden-onset hypernasality and fluid dysphagia. The condition is generally benign and self-limiting, with most cases resolving spontaneously or with minimal intervention, highlighting the importance of follow-up over aggressive treatment. Comprehensive diagnostic workups are essential to rule out more serious underlying conditions, and early speech therapy plays a crucial role in recovery. Continued documentation and further research are crucial to enhancing our understanding and improving management strategies for this rare disorder.
